# Wernicke's Encephalopathy in a Patient with Peptic Ulcer Disease

**DOI:** 10.1155/2011/156104

**Published:** 2011-06-28

**Authors:** Akinori Uruha, Toshio Shimizu, Tomoji Katoh, Yasushi Yamasaki, Shiro Matsubara

**Affiliations:** ^1^Department of Neurology, Tokyo Metropolitan Neurological Hospital, 2-6-1 Musashidai, Fuchu, Tokyo 183-0042, Japan; ^2^Department of Gastroenterology, Tokyo Metropolitan Tama Medical Center, 2-8-29 Musashidai, Fuchu, Tokyo 183-8524, Japan

## Abstract

We report a 74-year-old man with Wernicke's encephalopathy (WE) whose only prior illness was peptic ulcer disease. Upper gastrointestinal endoscopy demonstrated gastric ulcer scars accompanied by marked deformity, without pathologic evidence of malignancy. WE due to peptic ulcer disease in previous reports was substantially associated with thiamine deficiency due to recurrent vomiting or surgical procedures. In our case, however, there was no history of vomiting or gastrointestinal surgery. Besides, we thoroughly ruled out other known clinical settings related to WE. There is the possibility that peptic ulcer disease itself provoked thiamine deficiency due to malabsorption.

## 1. Introduction

Wernicke's encephalopathy (WE) is an acute neuropsychiatric syndrome characterized by nystagmus and ophthalmoplegia, ataxia of gait, and mental confusion. It results from thiamine (vitamin B1) deficiency and is observed mainly in alcoholics. Here, we describe a case of non-alcoholic WE in peptic ulcer disease. WE due to peptic ulcer disease in previous reports was substantially associated with thiamine deficiency due to recurrent vomiting or surgical procedures; the mechanisms of thiamine deficiency after surgical procedures include the occurrence of recurrent vomiting, poor compliance with an adequate dietary intake, and the reduced area of the gastrointestinal mucosa for absorbing thiamine [1, 2]. In our case, however, there was no history of vomiting or gastrointestinal surgery. We consider that peptic ulcer disease itself provoked thiamine deficiency due to malabsorption.

## 2. Case Report

**Figure 1:**
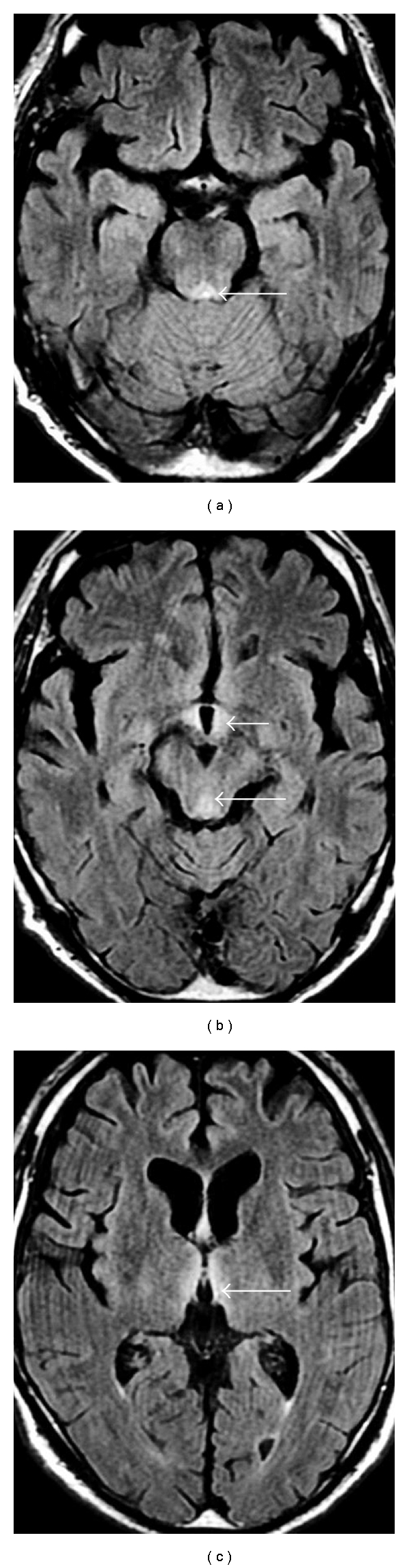
MRI-FLAIR images of the brain showed symmetrical high-intensity lesions (arrows) in the floor of the fourth ventricle (a), periaqueductal grey matter (b), hypothalami (b), and medial thalami (c).

**Figure 2:**
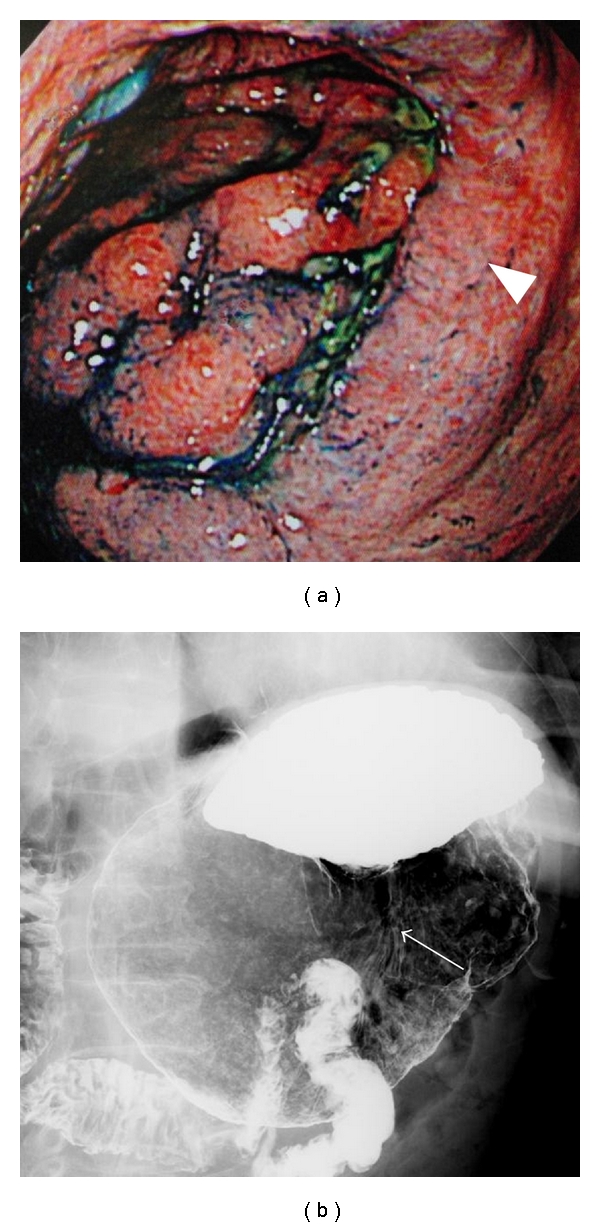
Marked deformity and shortening of the gastric angle (arrowhead) and lesser curvature (arrow), and insufficient distention of the pyloric antrum on upper gastrointestinal endoscopy (a) and gastric fluoroscopy (b).

A 74-year-old man was admitted to our hospital with subacute progression of diplopia, gait disturbance, and mental status change. He began to notice slight diplopia three months before admission, but he could work as a carpenter until several weeks before admission. According to his family members, he ate a balanced diet regularly and had not consumed alcohol in recent years. His past medical history was hemorrhagic gastric ulcer managed without surgery two years earlier, and then intake of lansoprazole was started. There was no history of vomiting lately. His family history was unremarkable. On admission, he exhibited mental sluggishness, disorientation, and anterograde amnesia. Neurological examination showed ophthalmoplegia, nystagmus, and ataxia. Blood tests demonstrated a low thiamine concentration (12.7 ng/mL; normal range 21.3–81.9 ng/mL). Results of other blood tests, including vitamins B2, B6, B12, folic acid, nicotinic acid, complete blood count, kidney and thyroid function, magnesium concentration, CEA, CA19-9, and IgG anti-*Helicobacter pylori* antibody, were normal. Brain magnetic resonance imaging (MRI) showed symmetrical high-intensity lesions in the medial thalami, hypothalami, periaqueductal grey matter, and the floor of the fourth ventricle on T2-weighted images and fluid-attenuated inversion recovery (FLAIR) images (Figure 1). Computed tomography of the chest and abdomen was unremarkable. Upper and lower gastrointestinal endoscopy demonstrated gastric ulcer scars accompanied by marked deformity (Figure 2). The duodenum and other parts of the gut were normal.

Intravenous thiamine (up to 1500 mg per day) was immediately administered for suspected WE on admission, and his consciousness, ocular manifestation and gait disturbance were markedly improved. However, disorientation and anterograde amnesia remained, and over the next several weeks he developed confabulation suggesting Korsakoff's syndrome. Multiple and repetitive biopsies of the gastric mucosa over the next two years showed chronic gastritis without evidence of malignancy.

## 3. Discussion

The present patient was diagnosed as having WE based on the low serum thiamine concentration, response of neurological signs to parenteral thiamine administration, and typical brain MRI findings. Clinical settings related to WE described in previous reports were thoroughly ruled out: chronic alcohol abuse and malnutrition; history of gastrointestinal surgical procedures; unbalanced nutrition including staple diet of polished rice; recurrent vomiting or chronic diarrhea; cancer; systemic diseases such as renal disease, hyperthyroidism, and chronic infectious febrile diseases; magnesium depletion; the use of drugs causing WE [1]. 

In general, thiamine is known to be absorbed in the duodenum. However, SLC19A2, one of the high-affinity thiamine transporters, was reported to show greater expression in the stomach more than in the duodenum [3]. This finding suggests that the stomach plays a more important role in thiamine absorption than previously thought. Thus, we consider that thiamine deficiency in this patient was due to thiamine malabsorption in the stomach involved with severe gastric mucosal lesions.

Patients with peptic ulcer disease often receive a proton-pump inhibitor (PPI). PPIs have been recognized to induce hypomagnesemia [4], which can provoke WE by suboptimum thiamine phosphorylation [5]. Although the use of PPIs should receive attention as a cause of WE, we do not consider in this respect that the PPI therapy was related to WE in our patient because the serum magnesium level was normal. Meanwhile, the mechanism of thiamine absorption in gastrointestinal lumen involves hydrogen cations, and the regular use of antacids may interfere with the absorption of thiamine though there has been no direct evidence [1]. Accordingly, we cannot completely deny the possibility that the long-term PPI use induced thiamine deficiency in this patient.

Since not every individual with a similar degree of thiamine deficiency develops WE [1], genetic factors may also be likely to contribute disease expression in our patient. Some studies have shown that variations in genes coding for transketolase and the high-affinity thiamine transporters are implicated in the pathophysiology of Wernicke-Korsakoff syndrome (WKS) [6–11]. In WKS, several genetic defects might combine with environmental factors to produce clinical manifestations when thiamine storage declines [1].

WE due to peptic ulcer disease in previous reports was substantially associated with thiamine deficiency due to recurrent vomiting or surgical procedures [1, 2]. This case is different in that respect, and there is the possibility that peptic ulcer disease itself provoked thiamine deficiency due to malabsorption. Indeed, it is unclear why only this case developed WE despite the large number of patients with peptic ulcer disease. However, clinicians should keep in mind that WE can occur as a rare complication of peptic ulcer disease because WE is a serious and preventable disease. We wish to emphasize that occasional measurement of blood thiamine levels is desirable in patients with severe gastric mucosal lesions.
